# Image-guided endoscopic marsupialization technique for frontal sinus mucocele with orbital extension: A case report

**DOI:** 10.1016/j.ijscr.2019.07.069

**Published:** 2019-07-31

**Authors:** Manuele Casale, Andrea Costantino, Lorenzo Sabatino, Michele Cassano, Antonio Moffa, Vittorio Rinaldi

**Affiliations:** a Department of Otolaryngology, Integrated Therapies in Otolaryngology, University Campus Bio-Medico, Rome, Italy; b Department of Otolaryngology, University of Foggia, Foggia, Italy

**Keywords:** Mucocele, Sinus surgery, Navigation system, Image-guided, Surgery, Case report

## Abstract

**Introduction:**

Frontal sinus mucocele with intra-orbital extension represents a rare benign cyst-like lesion. Surgical management could be summarized in an open approach, an endoscopic marsupialization or a combined procedure. The present study reports a case of frontal mucocele with wide intra-orbital invasion treated with endoscopic marsupialization assisted by an image-guided navigation system.

**Presentation of case:**

A 34-year-old African male was referred to the otolaryngology clinic for unilateral supraorbital swelling and post-nasal drip. A clinical ophthalmic assessment showed normal ocular movement, the absence of diplopia and normal visual acuity. CT scan showed a large soft tissue density lesion originating from the right frontal sinus with a supero-medial orbital erosion. The mass invaded the orbital cavity compressing and dislocating the eyeball forward and laterally. An image-guided ESS was performed according to Draft type IIa. Frontal mucocele’s inferior wall was open in order to drain muco-purulent content. No complications were detected and the patient was completely recovered with open frontal sinus drainage at 4 months follow-up visit.

**Discussion:**

We have successfully treated a wide intra-orbital frontal mucocele with an endoscopic marsupialization thanks to image-guided navigation system support. This technology prevented an external approach with associated morbidity and longer hospitalization.

**Conclusion:**

Navigated assisted endoscopic approach with marsupialization can be considered a safe treatment for FM with orbital extension. In particular, the image-guided system could be useful if bony landmarks are missing, if orbital erosion is present, and to completely drain lateral and multi-cystic lesions.

## Highlights

• Navigated assisted ESS is a safe treatment for FM with orbital extension.
• A navigation support could avoid an external approach.
• The image-guided system could be useful if bony landmarks are missing.
• The image-guided system could be useful if orbital erosion is present.
• Navigated assisted ESS could completely drain lateral and multi-cystic lesions.

## 1 Introduction

Frontal sinus mucocele (FM) represents a benign cyst-like lesion lined by the mucoperiostium of the frontal sinus cavity and filled with sterile mucus and shed epithelial cells [1]. Despite the benign nature of this condition, it deserves special attention for the capacity of expansion by virtue of a dynamic process of bone resorbation [2]. Although small FMs could remain asymptomatic for a long period, the primary clinical presentation could be represented by headache, facial deformity and nasal obstruction. If the FM invades the orbit, ophthalmic manifestations such as proptosis, impaired ocular mobility, and diplopia could occur. On the other hand, frontal sinus posterior wall erosion can lead to meningitis, meningoencephalitis, and cerebrospinal fluid fistula. Surgical management for FM could be an open approach (frontal sinus obliteration), an endoscopic marsupialization or a combined procedure. The chosen procedure mostly depends on the degree of the extension, surgical expertise and navigation system availability [3].

We report a case of a frontal mucocele with wide intra-orbital invasion which occurred in a young African male treated with endoscopic marsupialization assisted by an image-guided navigation system. This paper has been reported in line with SCARE criteria [4].

## 2 Presentation of case

**Fig. 1:**
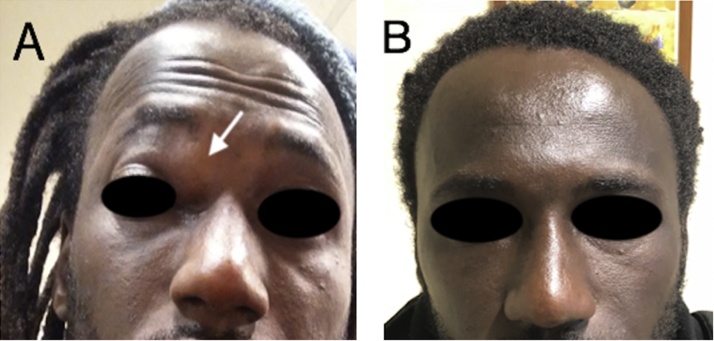
(A) Pre-operative right supraorbital swelling (white arrow) corresponding to frontal sinus mass. (B) Post-operative image of the successfully treated patient with no right supraorbital swelling.

A 34-year-old African male was referred to the otolaryngology clinic for unilateral (right) supraorbital swelling and post-nasal drip (Fig. 1A). The patient reported that the current clinical presentation has been present for the previous four years, but he was not treated as a consequence of poor health conditions and limited surgical equipment availability in his country. Diagnostic nasal endoscopy revealed edematous mucosa in the middle meatus of the nasal cavity.

The patient was then referred to a clinical ophthalmic evaluation that showed normal ocular movement and absence of diplopia for any gaze position associated with normal visual acuity.

**Fig. 2:**
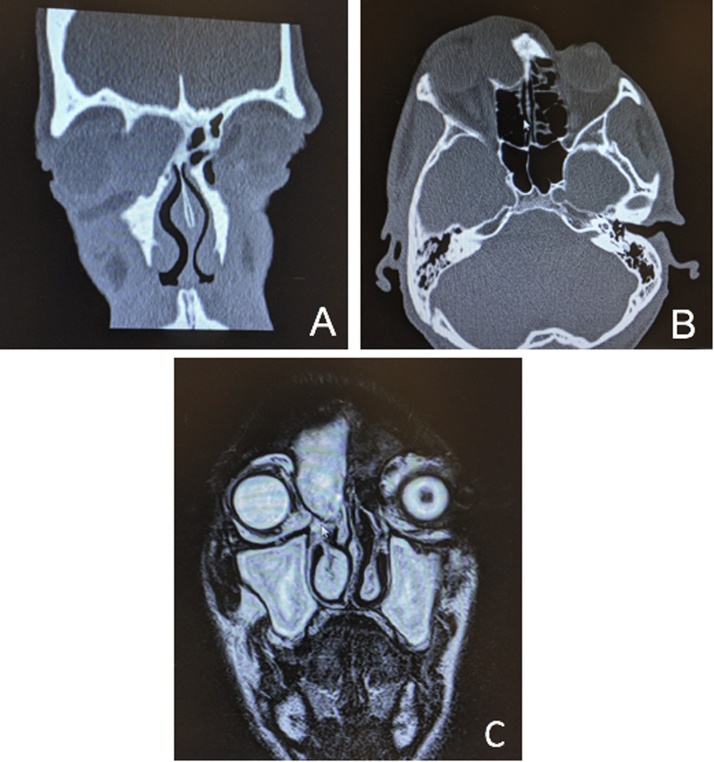
(A) Axial and (B) coronal plane of computed tomography scan showing a frontal sinus mucocele with intra-orbital extension. (C) Coronal view of contrast-enhanced magnetic resonance highlighting a benign fluid-filled lesion in the frontal sinus.

Computed tomography scan showed a large soft tissue density lesion (36 x 14 mm) in the right frontal sinus. Superior wall of the sinus was intact while the frontal sinus floor was eroded and the mass extended into the supero-medial aspect of the orbit (Fig. 2A-B). Then, a contrast-enhanced magnetic resonance (MR) confirmed the diagnosis of a benign fluid-filled lesion (Fig. 2C).

Prior to surgical procedure, the patient was treated with oral levofloxacin and corticosteroid for one week. An image-guided ESS was performed using the Medtronic® Fusion Compact™ ENT navigation system. After bilateral maxillary sinus mucoceles treatment with marsupialization and hyperplastic tissue removal, the frontal mucocele was approached. To better reach the fronto-ethmoidal recess and directly visualize the mucocele the frontal sinus floor was resected between lamina papyracea and the middle turbinate (Draft type IIa). Frontal mucocele’s inferior wall was open in order to drain muco-purulent content. At the end of the procedure the orbit appeared decompressed and the periorbita intact. No intra-operative or post-operative complications were detected and the patient was discharged the day after the procedure.

At 4 month follow-up visit the patient was completely recovered with open frontal sinus drainage (Fig. 1B). In addition, the patient was extremely satisfied with the clinical outcome, considering also some pre-operative aesthetic concerns about the open approach.

## 3 Discussion

The first paper describing the endoscopic marsupialization of sinus mucocele was published by Kennedy et al. in 1989 [5]. In the last 30 years we assisted an important improvement in endoscopic techniques and technologies leading to a significant improvement in FM surgical treatment. A recent meta-analysis confirmed the pivotal role of endoscopic sinus surgery (ESS) in the management of FM avoiding morbidity and longer hospitalization of the external approaches [3]. Several published case series have shown that endoscopic marsupialization has become the approach of choice in the majority of patients achieving an optimal clinical outcome with a lower recurrence rate [[6], [7], [8], [9], [10], [11]]. An open or combined approach for FM surgical management is almost required only for selected patients. Some authors suggest open surgery as a first line treatment in patients with unfavorable anatomy, such as case of underdeveloped sinus, a narrow anterior-posterior diameter of the frontal recess, a highly compartmentalized frontal sinus, a large, septated frontal sinus or with mucoceles that are situated within the lateral aspect of the frontal sinus [12]. In addition, an open approach is occasionally necessary as a salvage surgery in patients previously treated with ESS [13].

In this context, an-image guided ESS could represents the treatment of choice to avoid an open approach and the resulting complications. An image-guided navigation system allow the surgeon to perform surgical endoscopic procedure in a safer context thanks to a direct comparison of the intraoperative anatomy with preoperative imaging information. The surgeon may point to a specific structure during the procedure and then view the location of the instrument tip on the CT scan. This navigation system guide the ENT surgeon in surgical dissection preserving surrounding critical structures, such as the olfactory fossa, skull base, vascular structures (anterior ethmoid artery), and orbit [14]. Frontal sinus endoscopic surgery is one of the most technically demanding surgery regardless of surgical indication [15]. The complex anatomy of this region, frequently associated with inter-individual anatomical variability, and proximity to these critical structures, contribute to the technical difficulty of frontal recess surgery. In addition to traditional difficulties, FM determines several supplementary complexities during the procedure. First, a wide intra-orbital extension leads to confounding during surgical procedure because it is not easy to distinguish the FM from the periorbital soft tissue [9]. Second, a laterally extended FM could be difficult to reach in order to achieve a complete lesion marsupialization. In addition, FM could show internal septations determining the formation of a multi-cystic structure. For these reasons, we should carry out an extensive surgical debridement in order to reduce recurrence risk: the persistence of FM tissue after surgery could lead to the regrowth of the lesion and a consequent ESS failure. Finally, the growing of a FM disrupts the normal nasal bone anatomy making difficult to recognize fundamental anatomical landmark [8]. Because of these issues, an endoscopic approach could have been dangerous and potentially harmful. But nonetheless, we were able to safely perform ESS thanks to an image-based navigation system despite these several complexities related to this FM. The procedure was successfully completed, since we were able to entirely drain the FM, creating a huge sinus ostium in order to reduce recurrence risk.

## 4 Conclusion

Navigated assisted endoscopic approach with marsupialization can be considered a safe treatment for FM with orbital extension. In particular, the image-guided system could be useful if bony landmarks are missing, if orbital erosion is present, and to completely drain lateral and multi-cystic lesions.

## Funding

No funding was received for this research.

## Declaration of Competing Interest

All authors certify that they have no affiliations with or involvement in any organization or entity with any financial interest (such as honoraria; educational grants; participation in speakers’ bureaus; membership, employment, consultancies, stock ownership, or other equity interest; and expert testimony or patent-licensing arrangements), or non-financial interest (such as personal or professional relationships, affiliations, knowledge or beliefs) in the subject matter or materials discussed in this manuscript.

## Ethical approval

Ethical approval was not required and patient identifying knowledge was not presented in this report. Ethical approval is waived.

## Informed consent

Written informed consent was obtained from the patient for publication of this case report and accompanying images. A copy of the written consent is available for review by the Editor-in-Chief of this journal on request.

## Author contribution

All authors contributed significantly and in agreement with the content of the article.

Manuele Casale: Operating surgeon; Conceptualization; Methodology; Writing – Review & Editing.

Andrea Costantino: Conceptualization; Methodology; Writing – Original Draft; Writing – Review & Editing.

Lorenzo Sabatino: Operating surgeon; Data Curation.

Moffa Antonio: Writing – Original Draft.

Michele Cassano: Supervision.

Vittorio Rinaldi: Operating surgeon; Supervision.

## Registration of research studies

Not applicable.

## Guarantor

Andrea Costantino, MD.

## Provenance and peer review

Not commissioned, externally peer-reviewed.
